# Characterization of 3D printed biodegradable piezoelectric scaffolds for bone regeneration

**DOI:** 10.1002/cre2.712

**Published:** 2023-02-13

**Authors:** Divakar Karanth, David Puleo, Dolph Dawson, L. S. Holliday, Lina Sharab

**Affiliations:** ^1^ Department of Orthodontics University of Florida College of Dentistry Gainesville Florida USA; ^2^ Department of Biomedical Engineering University of Mississippi University Park Mississippi USA; ^3^ Department of Periodontics University of Kentucky College of Dentistry Lexington Kentucky USA; ^4^ Department of Orthodontics University of Kentucky College of Dentistry Lexington Kentucky USA

**Keywords:** 3D printing, bone regeneration, bone tissue engineering, poly(l‐lactic acid)

## Abstract

**Objective:**

The primary objective of this research was to develop a poly(l‐lactic acid) (PLLA) scaffold and evaluate critical characteristics essential for its biologic use as a craniofacial implant.

**Materials and Methods:**

PLLA scaffolds were designed and fabricated using fused deposition modeling technology. The surface morphology and microarchitecture were analyzed using scanning electron microscopy (SEM) and microCT, respectively. Crystallography, compressive modulus, and the piezoelectric potential generated upon mechanical distortion were characterized. Hydrolytic degradation was studied. MG63 osteoblast‐like cell proliferation and morphology were assessed.

**Results:**

The porosity of the scaffolds was 73%, with an average pore size of 450 µm and an average scaffold fiber thickness of 130 µm. The average compressive modulus was 244 MPa, and the scaffolds generated an electric potential of 25 mV upon cyclic/repeated loading. The crystallinity reduced from 27.5% to 13.9% during the 3D printing process. The hydrolytic degradation was minimal during a 12‐week period. Osteoblast‐like cells did not attach to the uncoated scaffold but attached well after coating the scaffold with fibrinogen. They then proliferated to cover the complete scaffold by Day 14.

**Conclusion:**

The PLLA scaffolds were designed and printed, proving the feasibility of 3D printing as a method of fabricating PLLA scaffolds. The elastic modulus was comparable to that of trabecular bone, and the piezoelectric properties of the PLLA were retained after 3D printing. The scaffolds were cytocompatible. These 3D‐printed PLLA scaffolds showed promising properties akin to the natural bone, and they warrant further investigation for bone regeneration.

## 1 INTRODUCTION

Physical bone defects can occur due to pathology, trauma, surgical resection of tumors, and maxillofacial reconstructive surgeries. They often require bone grafts to compensate for the lost tissue. Currently, bone graft options include autografts, allografts, or xenografts. However, autologous bone sources may involve considerable donor site morbidity, while allografts and xenografts may be compromised with immunological rejection (Cascalho & Platt, 2001). One bone tissue regeneration strategy involves using 3D scaffolds composed of biocompatible molecules to provide temporary support and guide cellular function.

An ideal bone tissue scaffold should be biocompatible and possess adequate mechanical properties to withstand functional forces. The bone scaffold should be osteoconductive and/or osteoinductive with a three‐dimensional lattice that facilitates blood vessel ingrowth and promotes the attachment, proliferation, and migration of osteoprogenitor cells. Osteoconductivity depends on the structural properties of the scaffold, such as fiber size, pore size, porosity, microarchitecture, particle size, and crystallinity (Cascalho & Platt, 2001). Scaffold can be seeded with the vital bone regenerating osteogenic stem cells that are capable of differentiating into osteoblasts and forming bone (Vo et al., 2012).

Bone dynamically remodels, guided by cellular and extracellular matrix signals, (Jacob et al., 2018) and bone collagen fibers are piezoelectric in nature, (Minary‐Jolandan & Yu, 2009) producing a streaming potential upon mechanical loading that is thought to assist in new bone tissue recruitment (Qin et al., 2002). This piezoelectric endogenous electric field discovery within biological tissues has fostered technological research into the use of biological electricity for tissue regeneration (Rajabi et al., 2015). Current synthetic osteoconductive/osteoinductive scaffolds typically do not generate or conduct electrical signals. Therefore, they may not support physiological piezoelectric pathways that guide cell growth and differentiation, (Kotwal, 2001) and osteoblast formation from stem cells. It would be advantageous for bone regeneration scaffold materials to not only conduct but also generate similar piezoelectric electric fields in response to mechanical stimulation.

Poly(l‐lactic acid) (PLLA) is approved by the US Food and Drug Administration (FDA) as a bone implant device (Ge et al., 2018). PLLA's mechanical properties enable orthopedic reconstruction; PLLA screws, pins, and plates are commonly used (Ashammakhi et al., 2001). In addition, PLLA is being explored by several research groups and it has been the most used material in 3D printing, even for biomedical use (Donate et al., 2020; Feng et al., 2021; Gregor et al., 2017; Hu et al., 2022; Oladapo et al., 2020; M. Wang et al., 2016). Rapid bone regeneration in some situations has been attributed to PLLA piezoelectric polarization. Ikada et al reported that implanted PLLA rods induced greater callus formation compared to polyethylene in feline tibiae over a 4‐ to 8‐week period. The authors attributed the enhanced results to possible PLLA piezoelectric generation induced by functional strain from the animals’ movements (Ikada et al., 1996).

The primary objective of this research was to develop a PLLA scaffold and evaluate critical characteristics essential for its biologic use for use as a craniofacial implant. These include the architecture of the 3D‐printed scaffolds, their hydrolytic degradation and piezoelectric properties, and their effects on cellular infiltration, attachment, and proliferation.

## 2 MATERIALS AND METHODS

### 2.1 Scaffold material, design, and development

Lactoprene® 100 M (Poly‐Med Inc) was used to fabricate the scaffold. Lactoprene® is a PLLA, 100% l‐lactide with a molecular weight of 165–170 kDa that is supplied as a 1.75 ± 0.05 mm diameter filament. Scaffold design was accomplished with design application software (Rhinoceros 5.0, Robert McNeel & Associates). The finished design included a fiber diameter of 150 μm and an osteogenic critical pore size of 450 µm. Multiple layers with these dimensions were stacked to obtain the desired overall size of the scaffold. Conversion of the standard tessellation language (STL) file to an applicable G code for the 3D print format was done with conversion software (PrusaSlicer 2.0, Prusa Research a.s., Praha, Czechia). Printing in these minute dimensions demanded a precise printer nozzle that could move without dragging the fiber from one point to another. G Code modifications controlled this issue and printed the scaffolds successfully. Scaffolds were fabricated with a fused deposition modeling printer (Prusa i3 MK3®, Prusa Research a.s.) equipped with an E3D v6 Nozzle (MatterHackers) with an inner diameter of 150 µm. The scaffolds were printed in the following configurations: 5 × 2 mm cylinders (*n* = 50); 5 × 10 mm cylinders (*n* = 10); 5 mm cubes (*n* = 6) and a 20 ×20 mm square sheet, one‐layer thick.

### 2.2 Scaffold characterization

#### 2.2.1 Surface morphology analysis

Samples were mounted on aluminum stubs (TedPella) and coated with a thin film of gold/palladium alloy using an EMScope SC400 sputter coating system at 20 µA for 1 min under argon gas. The shape and surface morphology of scaffolds were evaluated by scanning electron microscopy (SEM) (Quanta 250 FEG‐SEM, Thermo Fisher Scientific) using an accelerating voltage of 5–10 kV, at a working distance of 13.3–15.3 mm. Images were captured at several magnifications ranging from 57 to 668X. The average fiber thickness and average pore size were calculated with image processing software (ImageJ, version 1.52a, National Institutes of Health).

#### 2.2.2 Microarchitectural analysis

Scaffold microarchitectural morphology was obtained using a micro‐computerized tomography (microCT) scanner (µCT40, Scanco USA, Inc). Images were acquired with power settings of 70kVp, 114 µA, and 8 W scanning parameters and operating at high resolution (6 µm voxel size). Individual images were reconstructed into 3D models, and microarchitectural parameters such as porosity, pore distribution, and fiber and pore size were determined using the manufacturer's morphometry software (version 6).

#### 2.2.3 Crystallographic characterization

Scaffold PLLA crystalline content was estimated using X‐ray diffraction (XRD). Printed scaffold material was compared to PLLA filament to investigate any alterations in crystallinity caused by 3D printing. The crystallinity of “as supplied” PLLA polymer and three 3D‐printed scaffolds were obtained using conventional powder XRD (D8 Discover, Bruker AXS, Inc). Samples were scanned using CuKα radiation (*λ* = 1.54 Å; 40 mA, 40 kV) at room temperature in Bragg–Brentano configuration with 2θ = 2°–70°, with a step size of 0.02° and time/step of 20 s.

#### 2.2.4 Mechanical properties

Mechanical properties of scaffolds were determined using a Bose ElectroForce® 3300‐AT Series Test Instrument. The ElectroForce test instrument is integrated with a measurement transducer. A calibrated compressive force is applied to the sample at a strain rate of 0.5 mm/min. The software integrated with the machine plotted the relationship between stress and strain till the failure point. The mean elastic modulus was calculated from three scaffolds using the linear stress‐strain curve data between 0% and 2% of strain.

#### 2.2.5 Hydrolytic degradation

Nine 5 × 2 mm scaffolds were divided into groups of three and studied for either 4, 8 and 12 weeks. An additional three scaffolds were kept as controls and were stored dry in a freezer at −18**°**C. Initial dry weight measurements were made for all nine test samples and three controls. The control group scaffolds were weighed again at Week 4, 8 and 12. Test group scaffolds were immersed in 3 ml of phosphate‐buffered saline (PBS) in 6 ml screw‐top polyethylene vials sealed to prevent evaporation. These vials were placed on a compact orbital shaker in an incubator at 37**°**C, and the medium was changed every 72 h. At each time point, the scaffolds were extracted from the medium, rinsed in deionized water to remove the medium, dried, frozen at −50**°**C for 24 h, and lyophilized in a Labconco FreeZone Benchtop Freeze Dry System. The percentage of weight loss was calculated for each scaffold using the following formula The percentage of weight loss =  $\frac{\left(\text{Sample weight at baseline}\;–\;\text{Sample weight at}\;4,8,12\;\text{weeks}\right)}{\text{Sample weight at baseline}}$ *100

Furthermore, two scaffolds from each test group were evaluated using SEM, as described earlier.

#### 2.2.6 Piezoelectric property

Fine copper wire electrodes were attached to three scaffolds using conductive silver paint and stabilized with dental resin. The piezoelectric output under repeated cyclical loading and unloading was characterized using a Piezo film lab pre‐amplifier with 0.01–1000 mV/pC sensitivity range in charge mode and a Tektronix MDO3012 mixed domain oscilloscope.

### 2.3 Cytocompatibility

Human osteoblast‐like cells (MG63) were used to investigate the compatibility of the scaffolds (Filova et al., 2014). These MG63 cells were obtained from ATCC and cultured in the Eagle's minimum essential medium (EMEM) containing 10% fetal bovine serum. Before cell seeding, scaffolds were sterilized by immersion in 70% ethyl alcohol solution for 2 h, followed by exposure to ultraviolet light for 15 min on each side. To improve the attachment of cells on the scaffold, 15 µl of fibrinogen, human type I from human plasma (20 mg/ml in 0.9% saline; Sigma‐Aldrich) was placed on top of the scaffold. After 30 min, 15 µl thrombin from bovine plasma (50 U/ml in 40 mM CaCl_2_; Sigma‐Aldrich) was added. Five milliliter of 4 × 10^5^ cells/ml were added to each Petri plate with five scaffolds. The cells were incubated at 37**°**C in a 5% CO_2_ humidified incubator. The culture medium was replaced every other day. At 3, 7, 10, and 14 days of incubation, the scaffolds with cells were tested as follows.

#### 2.3.1 Cell proliferation assays

From the above‐mentioned cell culture, three scaffolds from each group (Days 3, 7, 10, and 14 group) were used to measure the proliferation of cells on the scaffold quantitatively using the Cell Counting Kit‐8 (CCK8) (Sigma‐Aldrich), according to the manufacturer's instructions. The amount of the formazan dye generated by the activities of dehydrogenases in cells is directly proportional to the number of living cells. At each time point, 500 µl CCK‐8 were added to the EMEM and 10% fetal bovine serum (FBS) and incubated for 2 h. The absorbance was detected at a wavelength of 450 nm by SpectraMax® M2 (Molecular Devices) multimode microplate readers. The results were analyzed using SoftMax® Pro Software.

#### 2.3.2 Cell attachment and cell morphology assessment

##### Scanning electron microscopy

Two scaffolds from each 3, 7, and 14 days groups were fixed in 2% glutaraldehyde for 2 h, washed with PBS, and dehydrated using ascending ethanol concentrations (e.g., 30, 50, 70, and 100%) for 30 min each, and then critical point dried. The scaffolds with cells were coated with a gold/palladium alloy thin film using an EMScope SC400 sputter coating as described earlier. MG63 cell attachment and morphology were qualitatively assessed under environmental SEM mode. (Quanta 250 FEG‐SEM, Thermo Fisher Scientific) using an accelerating voltage of 5–10 kV at a working distance of 13.3–15.3 mm.

##### Multiphoton microscopy (MPM)

Scaffolds with cells were washed in 1X PBS and then fixed in formaldehyde at room temperature for 30 min. They were washed in 1X PBS three times. The cytoskeleton of MG63 cells labeled using 1 µl of Phalloidin‐iFluor 488 Reagent in 1 ml of 1X PBS was added to the scaffold before the scaffold was then incubated at room temperature for 1 h. At 30 min, 1 µl/ml of DRAQ5™ Fluorescent Probe Solution (5 mM) was added to stain the nucleus. After staining, scaffolds were washed three times in 1X PBS. These procedures were performed on two scaffolds in each group (Days 3, 7, and 14). The three‐dimensional architecture of the cell growth and coverage on the scaffolds was observed using ZEISS Axio‐Examiner Z1 Confocal Laser scanning and multiphoton microscopy. The MPM system used in this study has been previously described in detail (S. Wang et al., 2019). In brief, the microscope is equipped with a Ti: sapphire laser (140 fs, 80 MHz), tunable from 690 to 1064 nm with an excitation wavelength of 810 nm. The average power after the objective was 5–10 mW. The emitted fluorescence was spectrally separated by passing it through a grating onto the 32‐channel gallium arsenide phosphide photomultiplier tube array detectors to get the SHG signal. The images were collected in two independent channels simultaneously: one channel covered the wavelength range from 395 to 415 nm for collection of SHG signals (color‐coded green), and the other channel covered the wavelength range from 430 to 690 nm for collection.

### 2.4 Statistical analysis

All data were analyzed using SAS 9.4® (SAS Institute, Inc). The mean and standard deviation (SD) were calculated. A Kolmogorov–Smirnov test confirmed normal data distribution with the mean data analyzed using one‐way‐analysis of variance with Tukey's post‐hoc test at a 95% level of confidence (*α* = .05).

## 3 RESULTS

### 3.1 Scaffold characterization

**Figure 1:**
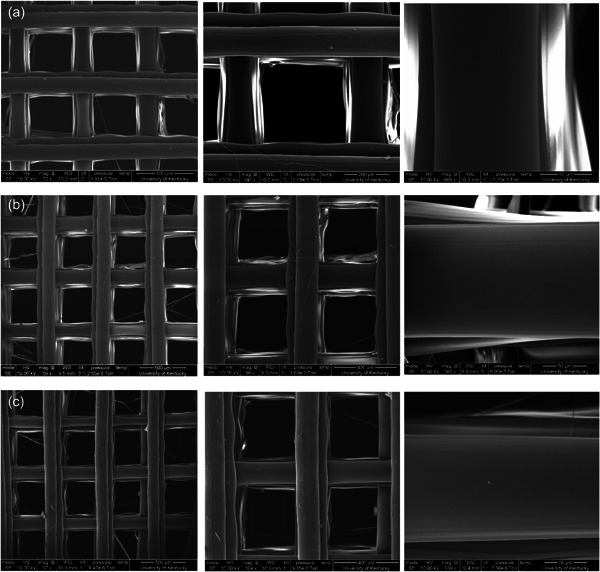
Scanning electron microscopy images showing the scaffold surface morphology: (a) Scaffold 1, (b) Scaffold 2, (c) Scaffold 3.

**Figure 2:**
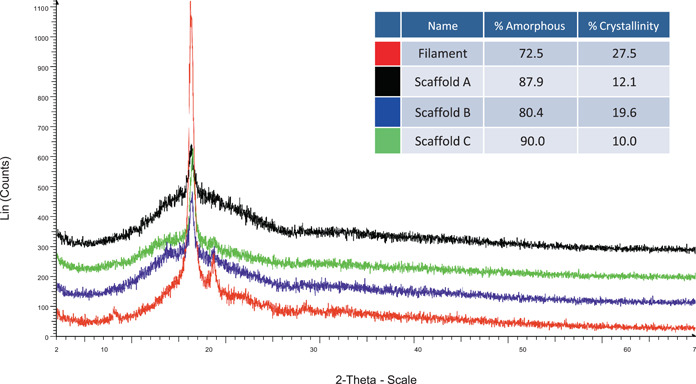
Overlay of powder diffraction data showing as‐supplied filament and 3D‐printed scaffolds.

SEM images visually revealed that the scaffold surface possessed a smooth texture (Figure 1). The mean fiber diameter was 130 ± 14 µm, while the mean pore size was 450 ± 13 μm. MicroCT analyses revealed an even distribution of the pores throughout the scaffold with a mean scaffold porosity of 73%. The as‐supplied PLLA filament was 27.5% crystalline, whereas the 3D‐printed scaffolds showed an average of 13.9 ± 5% crystallinity. The filament scan showed a small sharp peak present at around 16.5**°** 2‐theta on the broader peak, whereas the scaffolds showed a broader peak that started at ~5**°** 2‐theta and ended at around 27**°** 2‐theta (Figure 2). Mechanical testing of three scaffolds showed an average failure strain of 0.03 ± 0.003 and failure stress of 5.06 ± 1.48 MPa. From the stress‐strain data, the compressive modulus was calculated to be 244.3 ± 16.3 MPa.

The control and 4‐week groups did not show any significant weight changes, whereas the 8‐week and 12‐week groups showed an average weight loss of 1.5% and 0.7%, respectively. There was a statistically significant mean weight change in the 8‐week group. Analysis of the SEM images showed that the 4 and 8‐week scaffold fibers were thinner than the control groups by 5.9% and 15.2%, respectively.

**Figure 3:**
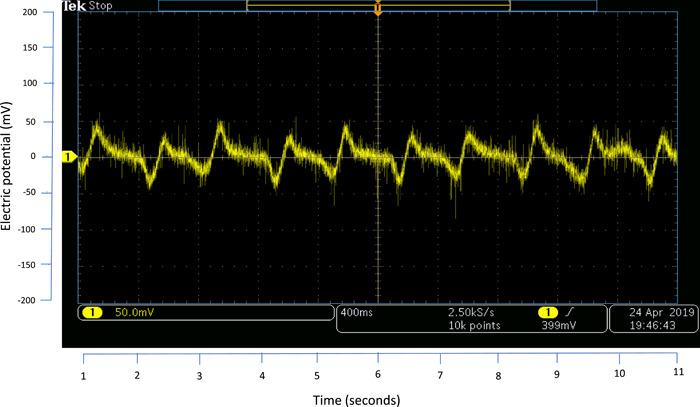
The piezoelectric potential is generated upon cyclic loading and unloading of the 3D‐printed poly(l‐lactic acid) scaffold.

The mean piezoelectric output generated was +25 mV upon loading the scaffold and −25 mV upon unloading. The repeated cyclical loading and unloading of 3 scaffolds showed consistent results (Figure 3).

**Figure 4:**
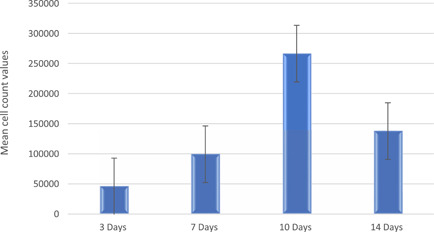
Number of MG63 cells on scaffolds on Days 3, 7, 10, and 14.

The number of cells present on the scaffolds was calculated based on the cellular dehydrogenase activity from the cell proliferation assays. The mean cell numbers ± SD were calculated to be 46,231 ± 710, 99,761 ± 145, 267,132 ± 173, and 138,248 ± 100 on Days 3, 7, 10, and 14, respectively (Figure 4). An increase in the viable cell number until Day 10 was statistically significant (*p* < .01). A decrease in the total number of metabolically active cells on Day 14 may be due to thick layers of cells made the deeper layer of cells devoid of nutrition. However, the microscopic evaluation showed cells grew in multiple layers on Day 14.

**Figure 5:**
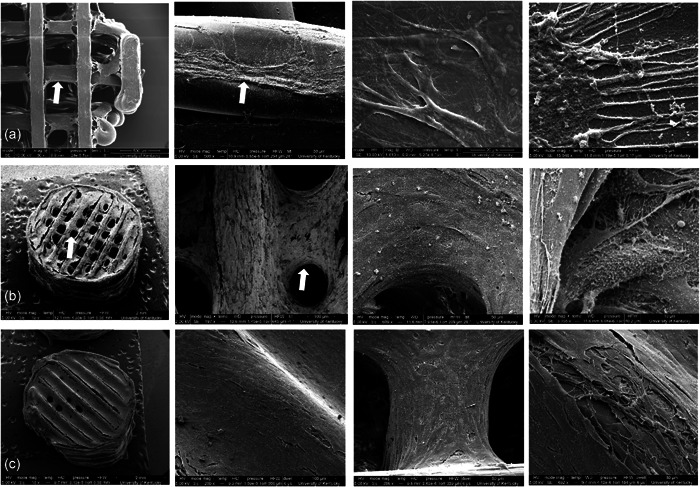
Scanning electron microscopy images showing cell‐seeded scaffolds: (a) Day 3, (b) Day 7, (c) Day 14. Arrows point to the cells attached to the filament of the scaffold and eventually spread to cover the pores.

**Figure 6:**
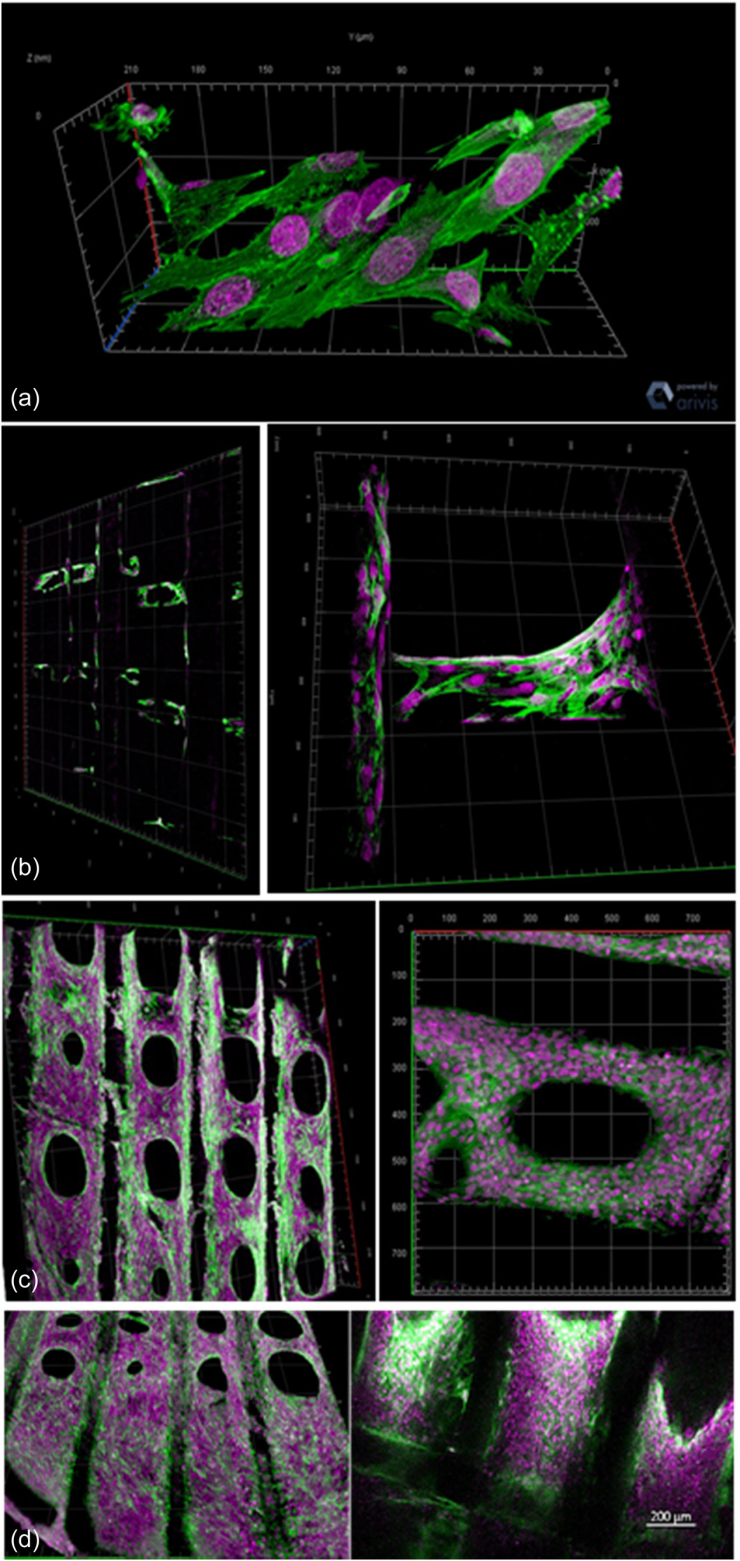
(a) Multiphoton microscopy images of MG63 cells, spindle‐shaped morphology typical for osteoblast cells with distinct cytoskeletal architecture. Actin filaments (green) were stained with CytoPainter Phalloidin‐iFluor 555 reagent (ab176755). DRAQ5 Fluorescent Probe used for staining the DNA content of the nucleus (purple). The cells on and within the pores of the scaffold on (b) Day 3, (c) Day 7, (d) Day 14. Arrows point to the cells attached to the filament of the scaffold and eventually spread to cover the pores.

MG‐63 cells had spindle‐shaped morphology when attached to the scaffold. Cell coverage increased as the experimental days progressed. On Day 14, cells grew in multiple layers and almost covered the pores of the scaffold (Figure 5). MPM evaluation showed similar findings on Day 3, when cells attached to the scaffold fibers showed typical osteoblastic morphology with a distinct cytoskeleton (Figure 6). Subsequently, cells spread from filament to filament across the pores. On Day 14, the cells entirely covered several pores in the scaffold (Figure 6).

## 4 DISCUSSION

Here we report the development of a piezoelectric PLLA scaffold that has characteristics that are compatible with bone regeneration. PLLA is an electroactive polymer with a piezoelectric coefficient similar to human bone (Fukada, 1998). This current research investigated whether the 3D printing process involving PLLA polymer melting and extrusion would alter the piezoelectric property of the polymer. Under the conditions of this study, repeat loading and unloading conditions generated an electrical potential between +25 and −25mV. These results were consistent for all scaffolds tested in this study. This proved that PLLA retained its piezoelectric characteristics after the 3D‐printing process.

PLLA crystallinity is an essential factor in piezoelectric properties (Tai et al., 2021). The crystallinity of the “as supplied” PLLA material was compared with the 3D‐printed material using an XRD powder Bragg‐Brentano method. Results show that some crystallinity content was lost in the 3D printing process. Melting, extrusion, and rapid cooling in the process of 3D printing were likely responsible for the reduced crystalline content of the polymer, confirming other reports (Mohseni et al., 2018). PLLA is a helical‐structured polymer, and its permanently oriented dipoles afford piezoelectricity generation (Fukada, 1992); the natural molecule orientation is sufficient to allow electrical generation (Jacob et al., 2018). The piezoelectric coefficient of PLLA (d14) is 10 pC/N, (Fukada, 1992; Rajabi et al., 2015) similar to the coefficient in human bone, which is reported to be between 7 and 12  pC/N (Halperin et al., 2004). The piezoelectric coefficient of compact bone is 0.1–0.3 pC/N (Rajabi et al., 2015) and of medullary bone, 8.48 pC/N (Halperin et al., 2004). In contrast, collagen's piezoelectric coefficient is 2 pC/N (Rajabi et al., 2015).

SEM images of the PLLA scaffolds in our study did not show any porosity on the surface except minute surface roughness. We found osteoblast‐like cells did not attach to uncoated PLLA scaffolds. However, after the scaffolds were coated with fibrinogen, and clotted with thrombin, as described by Sundararaj et al, (Sundararaj et al., 2015) the cells attached well. Although our use of fibrinogen was effective, other strategies might be explored, including oxygen plasma treatment (Park et al., 2016).

An advantage of 3D printing scaffolds is that the construct can be precisely sized, both at the macro level, so that it fits into a defect, but also at the micro level, to enable biocompatibility. Porous scaffolds facilitate an appropriate microenvironment for the migration, proliferation, and differentiation of cells and allow the mass transfer of nutrients, oxygen, and waste metabolic products within the structure (Velasco et al., 2015). Osteogenic cell proliferation and differentiation are affected by scaffold topography, pore geometry, and porosity (Huang et al., 2015; Von Recum & Van Kooten, 1996). Among the parameters of 3D scaffolds, pore size plays a vital role in supporting the cell ingrowth into the pores. A pore size of 100 μm is considered a minimum requirement for bone formation, (Itälä et al., 2001) with smaller pores supporting only fibrous tissue and osteochondral differentiation due to reduced blood vessel formation (Karageorgiou & Kaplan, 2005). For better vascularization and bone formation, however, scaffolds with 300 μm or larger pores are recommended (Karageorgiou & Kaplan, 2005). The average pore size of our scaffolds was 450 μm, which is favorable for cell penetration and proliferation. Gregor et al 3D printed the PLA scaffold using commercially available printers, concluding that standardization of layer size/filament diameter was diffcult (Gregor et al., 2017). The average porosity of the scaffolds fabricated in this research was 73%. When Lee et al scanned bone specimens from 30 human cadavers using high‐resolution micro‐CT, the alveolar trabecular bone had a porosity of 62% in D1 and 73% in D2 type bone (Lee et al., 2017).

Mechanical properties have been reported to correlate with scaffold porosity and scaffold fabrication methods. A bone replacement scaffold's mechanical property should allow withstanding bone functional axial loads. Mandibular trabecular bone (cortical plates present) has a reported compressive modulus ranging from 24.9 to 240 MPa, with a mean value of 96.2 ± 40.6 MPa (Misch et al., 1999). Bose and Roy found cancellous bone modulus ranged from 50 to 500 MPa, dependent on bone quality (Bose et al., 2012). Scaffolds in the current study possessed a compressive modulus of 244 ± 16 MPa. Serra et al reported the compressive modulus of 3D printed PLA‐based scaffolds in the range of 28 to 93 MPa (Serra et al., 2013). Donate et al used hydroxyapatite particles as an additive of the PLA matrix to increase the yield strength and Young's modulus (Donate et al., 2020).

Scaffold degradation is a critical factor in bone regeneration, as slow degradation delays and interferes with replacement bone formation and deposition. Contrastingly, rapid degradation can interfere with and lengthen the time it takes for bone formation. Polyesters, such as PLLA, undergo ester bond hydrolytic degradation during polymer erosion, (Tamai et al., 2000) producing nontoxic and water‐soluble lactic acid (Göpferich, 1996). However, too much lactic acid production can lead to inflammation and pain during the healing period (Rasal et al., 2010). Degradation rates depend on the material used, scaffold volume, scaffold porosity, physical environment, number of adjacent bony walls, patient age, and local vascularity (Wu & Ding, 2005). For example, more porous structures tend to degrade faster as more surface area is exposed to physiologic fluids. However, it is essential to ensure sufficient polymer exists during hydrolysis to withstand mechanical loading until bone deposition is complete. Hu et al suggested that by incorporating different proportions of inorganic materials, the PLLA degradation rate can be controlled in a targeted manner to cater to the demands of bone regeneration (Hu et al., 2022).

PLLA hydrolysis resistance is due to both the crystallinity and hydrophobicity of the polymer and in vitro bioresorption may take as long as two years, (Schumann et al., 2013) while clinical resorption may require over 3.5 years (Bergsma, 1995). PLLA has been used as a reconstructive material in maxillofacial osteosynthesis materials (Pihlajamaki et al., 1992). Commercial PLLA products such as GrandFix® (GUNZE) and FixsorbMX® (TEIJIN Medical Corp) have been used in maxillofacial bone surgery (Park, 2015; Sukegawa et al., 2016). Reported problems include foreign‐body reactions and late‐degradation tissue response (Bergsma et al., 1993; Bergsma, 1995). In additional biodegradation studies, Yoon and Kwon tested the biodegradation PLLA mesh in vivo and in vitro over 180 days, reporting no considerable in vitro weight and microstructure alteration as well as no significant change in the diameters of implanted mesh histologically (Yoon et al., 2017). de Tayrac et al implanted 15 PLA94 mesh samples in an incisional hernia Wistar rat model. Histopathology was performed up to 90 days after implantation. The water‐soluble degradation products appeared only after 8 months (de Tayrac et al., 2008). The 12‐week in vitro PLLA scaffold degradation observed in the present study displayed a small but statistically significant mass loss. The present experiment evaluated the short‐term degradation effect of a 3D‐printed PLLA scaffold. The SEM scaffold investigation did not reveal extensive surface erosion; however, it showed a decrease in fiber thickness compared to the control groups.

The Day 3 culture specimen SEM investigation showed MG63 cell attachment on the scaffolds with a typical flat spindle‐shaped phenotype and extended cytoplasm, both appearing to interact and associate with the adjacent cells. As time advanced, cells spread along the scaffold fibers and covered the entire scaffold surface by Day 7. Even though the sample preparation dehydration process caused a rupture of the thick cell layers of Day 14 samples, most of the scaffold pores were fully covered by the cells, as detected by SEM.

The MG63 cell's nuclei and F‐actin were labeled with fluorescent dyes to be observed by MPM. These images revealed the spindle‐shaped cell morphology typical for osteoblastic cells. The SEM morphological investigation and the MPM imaging showed excellent cell attachment to the scaffold. Gradually, these cells spread over the fibers, covering them in multiple layers and infiltrating the pores to reach the core regions of the scaffold. The scaffolds' 3D architecture favored the cell seeding within and around the scaffold and assisted in forming cell networks.

## 5 CONCLUSIONS

PLLA has been extensively explored in recent years for biomedical use. The current research validates the use of PLLA scaffolds for bone regeneration. PLLA scaffolds can be very accurately 3D printed per the design with an interconnected, porous microstructure. The mechanical and piezoelectric properties of these PLLA scaffolds were satisfactory. These scaffolds showed a prolonged rate of degradation. The cell attachment and proliferation on the scaffold proved the cytocompatibility of these 3D‐printed scaffolds.

3D‐printed PLLA scaffolds for bone regeneration showed promising results in this research. The technique of 3D printing with various scaffold designs for bone regeneration is worth investigating further. Additional series of experiments are necessary to answer all the questions thoroughly, such as in vivo animal experiments to study the host response to the PLLA scaffold, the scaffold's ability to regenerate bone, and biodegradability.

## AUTHOR CONTRIBUTIONS

Divakar Karanth conceptualized, designed, conducted the study, and drafted the manuscript. David Puleo supervised the research and provided the subject with expertise in interpreting the data. Dolph Dawson, L. S. Holliday, and Lina Sharab analyzed the data and critically reviewed the manuscript.

## CONFLICT OF INTEREST STATEMENT

The authors declare no conflict of interest.

## Data Availability

The data that support the findings of this study are available from the corresponding author upon reasonable request.
